# Prevalence of and Risk Factors for Sagittal Posture Abnormalities in Children Born With Esophageal Atresia: A Prospective Cohort Study

**DOI:** 10.3389/fped.2021.762078

**Published:** 2021-11-25

**Authors:** Benoit Bisson, Laurence Gottrand, Madeleine Aumar, Audrey Nicolas, Rony Sfeir, Julien Labreuche, André Thevenon, Frederic Gottrand

**Affiliations:** ^1^Univ. Lille, CHU Lille, Lille, France; ^2^IEM Dabbadie, APF France Handicap, Villeneuve d'Ascq, France; ^3^Univ. Lille, CHU Lille, Reference Centre for Chronic and Malformative Esophageal Diseases (CRACMO), Inserm U1286, Lille, France; ^4^Univ. Lille, CHU Lille, ULR 2694-METRICS, Évaluation des technologies de santé et des pratiques médicales, Lille, France

**Keywords:** esophageal atresia, VACTERL, kyphosis, sagittal postural patterns, children

## Abstract

**Introduction:** Scoliosis is a well-described complication of esophageal atresia (EA) caused by the associated spine malformations and/or thoracotomy. However, the sagittal posture abnormalities in patients with EA have not been described. The aim of this study was to evaluate the prevalence of and risk factors for sagittal posture abnormalities at the age of 6 years in patients operated on for EA.

**Methods:** A prospective cohort of 123 patients with EA was examined by the same rehabilitation doctor at the time of a multidisciplinary visit scheduled at the age of 6 years. Children presenting with scoliosis (*n* = 4) or who missed the consultation (*n* = 33) were excluded. Univariate and multivariate logistic regression models with Firth's penalized-likelihood approach were used to identify risk factors associated with sagittal posture anomalies. Candidate risk factors included neonatal characteristics, associated malformations, atresia type, postoperative complications, psychomotor development retardation, orthopedic abnormalities, and neurological hypotonia.

**Results:** The prevalence rates of sagittal posture abnormalities were 25.6% (*n* = 22; 95% CI, 16.7–36.1%). Multivariate analysis showed that minor orthopedic abnormalities (OR: 4.02, 95% CI: 1.29–13.43, *P* = 0.021), and VACTERL (OR: 3.35, 95% CI: 1.09–10.71, *P* = 0.042) were significant risk factors for sagittal posture abnormalities.

**Conclusion:** This study shows that sagittal posture anomalies occur frequently in children operated on at birth for EA and are not directly linked to the surgical repair. These children should be screened and treated using postural physiotherapy, especially those with VACTERL and minor orthopedic abnormalities.

## Introduction

Esophageal atresia (EA) is characterized anatomically by a congenital obstruction of the esophagus with interruption of the continuity of the esophageal wall and is caused by an abnormal embryological development. Congenital EA has an incidence of 1.8/10,000 births in France (1). The treatment for EA is surgery to close the esotracheal fistula, if present, and to restore esophageal continuity. The surgical procedure begins with a mini-invasive right posterolateral thoracotomy in the fourth intercostal space without costal resection or thoracoscopy followed by ligation of the tracheal fistula and anastomosis between the two pouches (2).

Musculoskeletal anomalies, mainly of the ribs and spine, occur in 50% of patients with EA. Scoliosis may result from surgery or be an associated congenital malformation (hemivertebrae). A recent population-based study reported a prevalence of 7.7% for thoracic malformations, including hemivertebrae, and that 58.1% (187/322) were the sequelae of surgery and included 85 costal hypoplasia, 47 other types of costal anomalies, 46 intercostal space anomalies, 21 costal fusions, and 12 cases of scoliosis (3). Another study from Canada found that scoliosis affected half of 106 patients who underwent thoracotomy for EA (4). The risk of scoliosis was 13-fold after repair of EA compared with the general population (5). Rib cage deformation (chest wall asymmetry, winged scapula) was reported in 20% of patients and scoliosis in 10% of patients (6).

Although scoliosis is a well-described complication of EA, sagittal posture abnormalities (SPA) have never been studied in patients with EA. Although it does not cause significant breathing consequences, the accentuation of the curvature can lead to back pain and an increased risk of pathological kyphosis and stiffening of the spine. The aim of our study was to assess the prevalence of and risk factors for SPA at 6 years of age in patients with EA.

## Method

### Patients

In the framework of the National Plan for Rare Diseases (7) and based on each patient's progress, all children with EA have a systematic visit at a multidisciplinary outpatient consultation, including a complete musculoskeletal examination, at the age of 6 years. We took this opportunity to study SPA prospectively in patients starting at the age of 6 years. The same experienced rehabilitation doctor (LG) examined these children from 2009 to 2019. All children who were admitted to the physical medicine and rehabilitation outpatient clinic at the age of 6 years during this period were included. Because SPA are more frequent in patients with scoliosis, we excluded children with scoliosis confirmed on radiography.

### Data Collection

**Figure 1:**
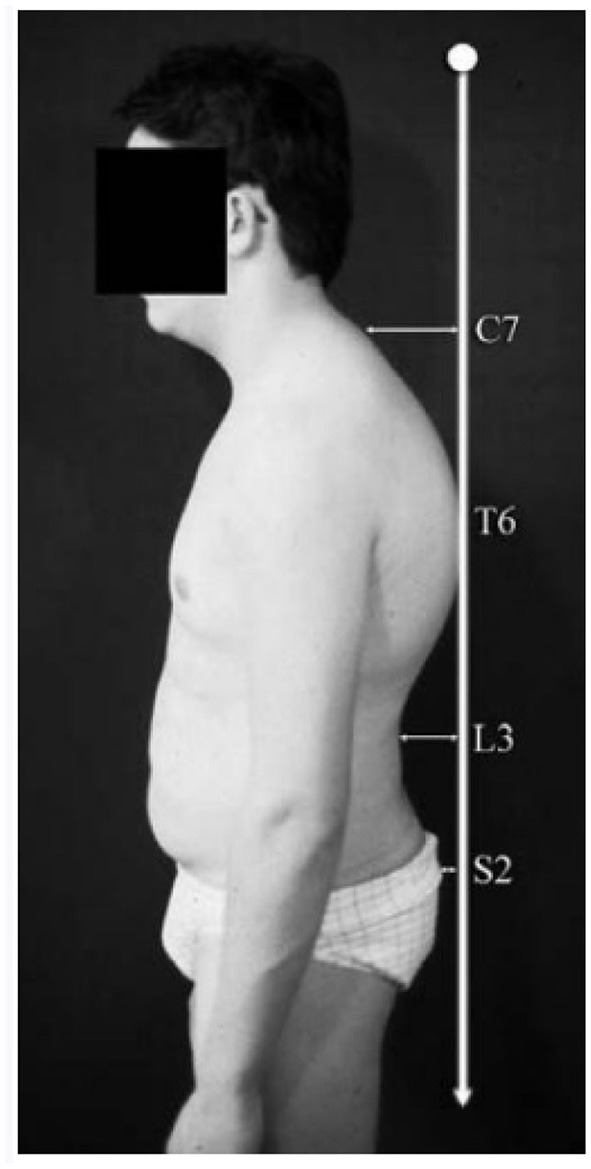
Spinal arrows measurement.

**Figure 2:**
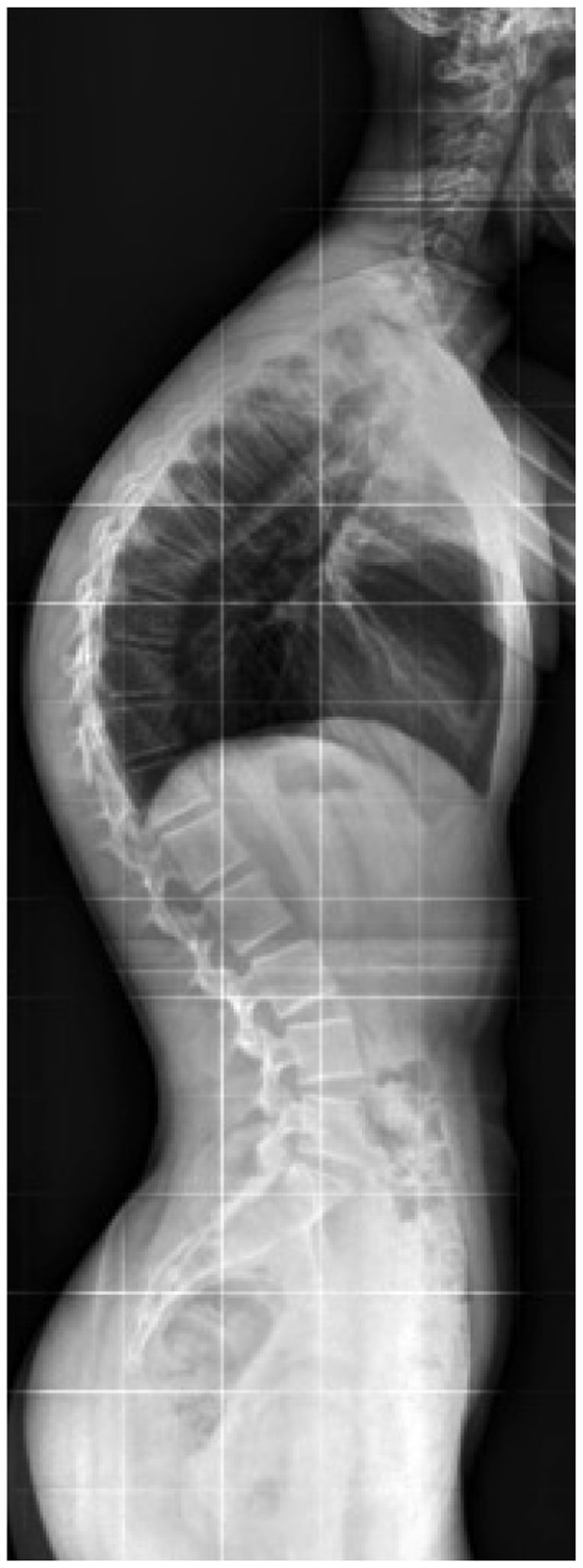
Profile x-ray of the spine.

Kyphosis is a sagittal disorder of the spine, and its diagnosis is based clinically on the arrow of kyphosis measured with plumb lines measured using the half sum of the high thoracic arrow measured at C7 and the lumbar lordosis arrow at L2 (Figure 1). We defined kyphosis as an arrow >40 mm. SPA can be corrected actively or passively, although kyphosis is a definite deformation (Figure 2). For clarity, we use the term “kyphosis” here as a generic term that includes all types of SPA.

The following risk factors were studied: neonatal characteristics; sex; prematurity (<37 weeks of amenorrhea); intrauterine growth retardation (defined as a neonate of size and/or weight <10th percentile for gestational age); associated malformations (VACTERL or CHARGE); atresia type (with or without esotracheal fistula following the Ladd classification); time in relation to surgery (defined as early if <15 days or equal, late if more than 15 days); postoperative complications (all combined); esophageal anastomotic stricture (defined as a reduction in esophageal diameter and clinical signs) (8); tracheomalacia; existence of a gastrostomy; history of abdominal surgery; psychomotor developmental delay (defined as the lack of acquisition of head posture at 4 months, sitting at 9 months, or walking at 18 months); orthopedic abnormalities (in general) or minor orthopedic abnormalities (defined as disorders of the structure of the lower limbs: genu valgum, excessive anterior femoral torsion, flat feet, hollow feet); other congenital malformations; scar problems (keloid or inflammatory scars, or adherent scarring); dyskinesia of the scapula (corresponding to the abnormal movements of the scapula: winged scapula, abnormal tilting of the scapula); and neurological hypotonia relative to age and development.

Postoperative complications were recorded and included anastomotic stricture, infection, anastomotic leak, tracheomalacia, and gastroesophageal reflux disease, which was defined as peptic esophagitis at the time of endoscopy, abnormal pH/impedancemetry, and/or typical clinical signs.

### Statistical Analysis

All variables are categorical and are described as number (percentage). The prevalence of SPA was estimated by calculating the 95% confidence interval (CIs) using the Clopper–Pearson method. Univariate and multivariate logistic regression models were used to identify the risk factors for SPA using Firth's penalized-likelihood approach to account for the small sample size (9). Factors associated with SPA in univariate analysis (at *P* < 0.10) were added as candidate variables to the multivariate analysis using a backward-stepwise selection procedure with *P* < 0.10 as the selection criterion. Before developing the multivariate model, we examined the absence of collinearity between the candidate predictors by calculating the variance inflation factors (10). Odds ratios (ORs) and their 95% CIs were derived as effect sizes from logistic models. We examined the performance of the selected models in terms of calibration using the Hosmer–Lemeshow goodness-of-fit test and discrimination by calculating the c-statistics (11). Statistical testing was performed at the two-tailed α level of 0.05. Data were analyzed using SAS software (version 9.4, SAS Institute Inc., Cary, NC, USA).

### Ethics

The study was approved by the Ethical Committee of the French-speaking Group of Gastroenterology-Hepatology and Pediatric Nutrition (N° 2020-021). All data were used anonymously, and the parents were informed of the aims of the study and were asked if they agreed to participate.

## Results

During the study period, 123 patients with EA were followed up in our center, and 37 patients were excluded: 33 patients who did not return to the physical medicine and rehabilitation consultation at 6 years of age and 4 patients who had scoliosis. Our study included 86 patients or 70% of the eligible population. The sample was 63% boys (*n* = 54); 88% had an esotracheal fistula, 8% had long-gap EA (type I), and 60% exhibited postoperative complications including anastomotic stricture in 42% and tracheomalacia in 25.6%. Scar problems were rare (Table 1).

**Table 1:** Characteristics of the 86 Infants with EA who had a physical medicine and rehabilitation consultation at 6 years of age.

**Characteristic**	**Value**
Boys	54/86 (62.8)
Prematurity	35/86 (41.2)
Intrauterine growth retardation	32/86 (38)
**Atresia type**	
Type I	7/86 (8.1)
Type III	76/86 (88.4)
Type IV	2/86 (2.3)
Type V	1/86 (1.2)
VACTERL	19/86 (22.1)
**Time of surgery**	
Before and day 15	79/86 (91.9)
After day 15	7/86 (8.1)
Postoperative complications	52/86 (60.5)
Esophageal stenosis	36/86 (41.9)
Tracheomalacia	22/86 (25.6)
Gastrostomy	22/86 (25.6)
History of abdominal surgery	22/86 (25.6)
Psychomotor development retardation	19/86 (22.1)
Orthopedic abnormality	48/86 (55.8)
Congenital malformation	24/86 (27.9)
Minor orthopedic abnormalities	23/86 (26.7)
Scar problem	7/86 (8.1)
Dyskinesia of the scapula	24/86 (27.9)
Kyphosis	22/86 (25.6)
Hypotonia	10/86 (11.6)

*Values are expressed as the number/total number of patients (%)*.

The prevalence of SPA was 25.6% (*n* = 22; 95% CI, 16.7–36.1). The prevalence of SPA did not differ significantly between girls (*n* = 9, 28.1%; 95% CI, 13.7–46.8) and boys (*n* = 13, 24.1%; 95% CI, 13.4–37.7).

In the univariate analysis, the presence of SPA was significantly associated with VACTERL, psychomotor development delay, minor orthopedic abnormalities, and hypotonia but was not associated with tracheomalacia, history of abdominal surgery, or orthopedic abnormalities (Table 2). In the multivariate analysis, only minor orthopedic abnormalities and VACTERL were associated with SPA, whereas tracheomalacia and hypotonia did not reach significance (Table 3). This multivariate model had good discrimination (c-statistic = 0.76) and no deviation in calibration as indicated by the Hosmer–Lemeshow goodness-of-fit test (*P* = 0.22).

**Table 2:** Factors associated with sagittal posture abnormalities in the univariate analysis.

	**Sagittal posture abnormalities**			
**Factor**	**No (*n* = 64)**	**Yes (*n* = 22)**	**OR (95% CI)**	***P-*value**
Boys	41/64 (64.1)	13/22 (59.1)	0.81 (0.30–2.18)	0.67
Prematurity	27/63 (42.9)	8/22 (36.4)	0.78 (0.28–2.05)	0.62
Intrauterine growth Retardation	22/63 (34.9)	10/22 (45.5)	1.55 (0.58–4.10)	0.38
VACTERL	10/64 (15.6)	9/22 (40.9)	**3.65 (1.26–10.72)**	**0.019**
Postoperative complication	36/64 (56.3)	15/22 (68.2)	1.61 (0.60–4.59)	0.35
Esophageal stenosis	25/64 (39.1)	11/22 (50.0)	1.55 (0.59–4.08)	0.38
Tracheomalacia	13/64 (20.3)	9/22 (40.9)	2.69 (0.95–7.53)	0.063
Gastrostomy	16/64 (25.0)	6/22 (27.3)	1.16 (0.38–3.29)	0.79
History of abdominal surgery	13/64 (20.3)	9/22 (40.9)	2.69 (0.95–7.53)	0.063
Psychomotor development delay	10/64 (15.6)	9/22 (40.9)	**3.65 (1.26–10.73)**	**0.019**
Orthopedic abnormality	32/64 (50.0)	16/22 (72.7)	2.54 (0.93–7.56)	0.080
Congenital malformation	16/64 (25.0)	8/22 (36.4)	1.72 (0.61–4.73)	0.31
Minor orthopedic abnormality	13/64 (20.3)	10/22 (45.5)	**3.20 (1.15–8.98)**	**0.027**
Dyskinesia of the scapula	15/64 (23.4)	9/22 (40.9)	2.25 (0.81–6.18)	0.12
Hypotonia	4/64 (6.3)	6/22 (27.3)	**5.30 (1.43–21.28)**	**0.017**

*Values are expressed as number/total number (%) unless indicated otherwise*.

*OR, odds ratio; CI, confidence interval*.

*The bold values are the significant results of the univariate analysis (factors associated with kyphosis)*.

**Table 3:** Factors associated with sagittal posture abnormalities in the multivariate analysis.

**Factor**	**OR (95% CI)**	***P*-value**
Minor orthopedic abnormalities	**4.02 (1.29–13.43)**	**0.02**
VACTERL	**3.35 (1.09–10.71)**	**0.04**
Tracheomalacia	3.14 (0.96–10.47)	0.07
Hypotonia	3.51 (0.86–15.08)	0.1
*c-statistic (95% CI)*	0.76 (0.63–0.90)	
*Goodness-of-fit test*	*P*-value = 0.22	

*ORs were calculated using a backward-stepwise multivariable penalized-likelihood logistic model. Candidate predictors were VACTERL, tracheomalacia, history of abdominal surgery, psychomotor development delay, orthopedic abnormality, minor orthopedic abnormalities, and hypotonia. The bold values are are the significant results of the multivariate analysis (factors associated with kyphosis)*.

## Discussion

This is the first study to analyze SPA in children operated on for EA. We found a high prevalence of this complication in children who underwent a mini-invasive open right thoracotomy. Although we did not have a control group to compare the prevalence of kyphosis in children without EA, the published prevalence of kyphosis in the general pediatric population is half that of children with EA. For example, in a study of five schools in Germany that included 2,075 children aged 10–17 years, Nitzschke et al. found a prevalence of kyphosis of 15.3% for boys and 12% for girls (12). This high prevalence supports the idea that patients with EA are at high risk of kyphosis and, therefore, require systematic screening.

The only two factors we found associated with kyphosis were minor orthopedic abnormalities and VACTERL, whereas neither the EA type nor surgical issues and complications were associated with sagittal disorders.

Most of the minor orthopedic anomalies involved excessive femoral antetorsion and flat valgus feet. These two findings have little or no functional impact, and these conditions develop favorably by the end of growth. Their association with kyphosis suggests that EA is associated with benign orthopedic problems besides scoliosis and bone malformations.

As expected, kyphosis was associated with VACTERL, a complex malformation that involves vertebral and costal malformations. However, when analyzing the variable “congenital malformation,” which included vertebral, costal malformations, and other malformations, we did not find a significant association with kyphosis. The VACTERL syndrome is associated with kyphosis beside scoliosis (13). Vertebral anomalies, which are commonly accompanied by rib anomalies, have been reported in 60–80% of patients with VACTERL syndrome (14). Interestingly, patients may have rib anomalies without vertebral anomalies. Vertebral anomalies typically include segmentation defects, such as hemivertebrae, “butterfly vertebrae,” “wedge vertebrae,” or vertebral fusions, supernumerary or absent vertebrae, and other forms of vertebral dysplasia. A wide range of severity has been reported for vertebral malformations, and some patients may have subtle defects that are detectable only through careful scrutiny by an experienced clinician.

We were surprised not to find a relationship between hypotonia, prematurity, and kyphosis. Because tone increases with gestational age, preterm infants are more prone to develop neurological hypotonia. White matter abnormalities of the corpus callosum have been reported in both premature and full-term infants with long-gap (type I) EA (15). Moreover, brain volume is decreased in these children, which implies delayed brain growth (16). Brain volume is an important predictor of neurodevelopmental outcomes in premature babies, although the neurodevelopmental consequence of EA remains to be studied. A recent retrospective study that assessed the neurodevelopmental outcome of 253 infants with EA at the age of 12–36 months (*n* = 182) reported that 76% were within the normal range, although some delay was seen in 24% of the infants, most often in expressive and receptive language (17). However, most of the patients with kyphosis in our study had normal neurological examination results.

One important and controversial question in EA patients is the effects on outcomes of the surgical technique, such as thoracotomy, mini-invasive thoracotomy, or thoracoscopy. Although a “historical” thoracotomy that includes muscle and costal damage has a negative effect on thoracic development and scoliosis (14, 18), the advantages of the thoracoscopic vs. mini-invasive thoracotomy in terms of orthopedic sequelae remain to be established. A systematic review of 25 recent studies (19) concluded that, when conducted by experienced surgeons, thoracoscopic repair is a safe and minimally invasive alternative to conventional open repair and may yield better results in appropriately selected patients. However, this systematic review did not address the impact on scoliosis or long-term effects. All patients in our study underwent EA repair using a mini-invasive right thoracotomy technique, and we could not compare the different surgical techniques in terms of their effects on scoliosis or kyphosis. Given that anastomotic delay and scar problems were very rare, it was not possible to study them as potential risk factors.

Our study has strengths and limitations. Because of the relative small sample size and limited number of SPA (*n* = 22), we note that our study may not have adequate statistical power to have assessed all factors associated with the presence of SPA. We cannot exclude the possibility that some associations that were significant in the univariate analyses may have been overlooked and were not included in the multivariate analysis to identify independent predictors. A strength of our study is that it was a large prospective study in which all patients were examined by the same rehabilitation doctor following a systematic protocol of SPA evaluation.

Our findings suggest that children operated on for EA, particularly those with minor foot and knee orthopedic problems and VACTERL syndrome, should be screened for SPA. It is important that screening occurs before the onset of spine growth because spinal deformities may worsen after this point. A child begins to acquire the adult morphotype from the age of 5 years (20). We chose the age of 6 years because psychomotor skills are present and allow for a reliable examination.

Screening can be clinical or by using innovative imaging techniques that allow external measurements of the spinal curvature without irradiation. The distribution of these devices remains limited and they are not yet available in everyday practice.

Screening for SPA is important because they can be corrected using active physiotherapy reeducation together with learning spinal self-exercises and techniques. Self-exercises must be repeated daily and integrated into complementary sport activities and activities of daily living (21). Physiotherapy should focus on static bodybuilding in active axial elongation combined with breathing exercises with an emphasis on deep inspiration. The second focus of rehabilitation is based on proprioceptive control (21). Patients with non-reducible kyphosis require both reeducative work as well as orthopedic care (brace) or surgical intervention, primarily for the management of angular kyphosis (vertebral arthrodesis). In our study, no children required surgical treatment.

## Conclusion

Given the high prevalence of kyphosis in children with EA, screening and follow-up by a specialist in physical medicine and rehabilitation is needed. The age of 6 years seems to be the ideal age for medical consultation to detect and manage disorders before adolescence. Special attention should be paid to children with VACTERL and minor orthopedic problems.

## Data Availability Statement

The raw data supporting the conclusions of this article will be made available by the authors, without undue reservation.

## Ethics Statement

The studies involving human participants were reviewed and approved by Groupe Francophone d'Hépatologie - Gastroentérologie et Nutrition Pédiatrique. Written informed consent from the participants' legal guardian/next of kin was not required to participate in this study in accordance with the national legislation and the institutional requirements.

## Author Contributions

LG and FG contributed to conception and design of the study. LG, MA, AN, RS, and FG organized the database. BB wrote the first draft of the manuscript. JL performed the statistical analysis. AT, FG, and LG contributed to manuscript revision, read, and approved the submitted version. All authors contributed to the article and approved the submitted version.

## Conflict of Interest

The authors declare that the research was conducted in the absence of any commercial or financial relationships that could be construed as a potential conflict of interest.

## Publisher's Note

All claims expressed in this article are solely those of the authors and do not necessarily represent those of their affiliated organizations, or those of the publisher, the editors and the reviewers. Any product that may be evaluated in this article, or claim that may be made by its manufacturer, is not guaranteed or endorsed by the publisher.

## Abbreviations

EA: Esophageal atresia
SPA: Sagittal posture abnormalities.
